# Nutrition Transition and Traditional Food Cultural Changes in Sri Lanka during Colonization and Post-Colonization

**DOI:** 10.3390/foods7070111

**Published:** 2018-07-13

**Authors:** Permani C. Weerasekara, Chandana R. Withanachchi, G. A. S. Ginigaddara, Angelika Ploeger

**Affiliations:** 1 Section of Organic Food Quality and Food Culture, Faculty of Organic Agricultural Sciences, University of Kassel, 34125 Kassel, Germany; a.ploeger@uni-kassel.de; 2 Department of Archaeology and Heritage Management, Faculty of Social Sciences and Humanities, Rajarata University of Sri Lanka, Anuradhapura 50000, Sri Lanka; chandanawithanachchi@gmail.com; 3 Department of Agricultural Systems, Faculty of Agriculture, Rajarata University of Sri Lanka, Anuradhapura 50000, Sri Lanka; sanjeewanieg@gmail.com

**Keywords:** food transition, food habit, westernization, dietary patterns, health impact

## Abstract

Sri Lanka was a colony of the Portuguese, Dutch, and British. The simplification of Sri Lankan food culture can be seen most clearly today, including how the diet has been changed in the last 400 years since the colonial occupation began. Therefore, greater efforts must be made to uncover the colonial forces that have undermined food security and health in Sri Lanka. Also traditional eating habits, which are associated with countless health benefits, have been gradually replaced by the globalized food system of multinational corporations and hidden hunger, a system inherent in the emergence of non-communicable diseases, such as cancer, diabetes, cholesterol, and kidney disease epidemics, in Sri Lanka. This article discusses factors that have underpinned the dietary change in Sri Lanka from its early colonization to the post-colonization period. The research followed the integrated concept in ethnological and sociological study approaches. The study examined literature and conducted several interviews with field experts and senior people in marginal areas in Sri Lanka. This study examines the Sri Lankan traditional food system and how it changed after the colonial period, including the main changes and their impact on current micronutrient deficiencies and non-communicable diseases.

## 1. Introduction

Today, the estimated world population of undernourished people with respect to dietary energy supply is almost 842 million [1]. This persists globally, even though there is enough food for all. Furthermore, 26% of children experience stunted growth, and 30% of the world’s population suffers from micronutrient deficiencies. This issue is particularly critical in developing countries, which account for nearly 98% of the world’s chronically hungry people [1]. According to the United Nations Children’s Fund (UNICEF) report (2017) for South Asia, about 39% of children under the age of five have experienced stunted growth, and nearly three-quarter of people with micronutrient deficiencies live in Asia [2]. In Sri Lanka, nearly every fourth (22%) pregnant woman is considered underweight at the time of registration for pregnancy. This increases the risk of intrauterine growth retardation and low birth weight. More than one in five households are affected by food insecurity, which is defined as unreliable access to calories [1,2,3]. However, many families are unlikely to have access to a variety of diets throughout the year. Micronutrient deficiencies are common in populations who consume poor-quality diets lacking diversity. Vitamin A deficiency accounts for 29.3% of children in Sri Lanka, while 2.3% of these children have a severe deficiency. A total of 35% of pre-school children and 23% of pregnant women have a vitamin A deficiency [1]. Nearly 15% of mothers with children from 6 to 60 months of age have vitamin A deficiency (1). Also, non-communicable diseases (NCDs) are increasing day by day. Sri Lanka has been witnessing in the past few decades a rapid upsurge of NCDs, which includes epidemics of diabetes, various cancers, and increased blood cholesterol levels, claiming 103,500 lives each year. In general, 75% of all deaths are due to NCDs in Sri Lanka [1,4,5]. These are all problems related to food habits. Therefore, food transition could be considered to be a severe public problem within the country.

The fundamental issues to be dealt with regarding food transition in Sri Lanka involve income-induced diet diversification, dietary globalization, and Westernization. Westernization is a process by which societies come under or adopt the Western culture in areas such as industry, technology, law, politics, economics, lifestyle, diet, cloth, language, religion, philosophy, and values [6]. As urbanization and globalization begin to exert their influence, we can see the adoption of markedly different diets that no longer conform to traditional local habits. The new dietary habits reflect Western patterns and could be quite unlike the habits that developed locally over many generations. Large urban markets have space for establishing a supermarket chain as they attract foreign investment and advertising from global corporations [7]. The traditional food supply chain cannot meet the rapid diversification of the diet. It requires, in effect, the modernization of the food retail sector and the vertical integration of the food supply chain, including the diversification of agricultural systems in Sri Lanka. The above quantitative, qualitative, and organizational changes in the urban food supply drive the processes of commercialization and diversification of domestic production systems [7]. However, the nutrition transition is not just a result of the growth of supermarkets, as research has shown the wide availability of processed and unhealthy foods in modern retail in Sri Lanka [6]. Thus, it is a result of colonization. Not only colonial policies but also their cultural values and lifestyle patterns, have directly and indirectly affected the Sri Lankan lifestyle. 

Since the 1500s, when the imperial powers of Europe sought to expand their empires through the colonization of Sri Lanka, the presence of ancient indigenous knowledge, including an incredible wealth of experience about food habits, health, and longevity, has progressively waned. Food was always a fundamental tool in the process of colonization. Colonization is a violent process that fundamentally altered the ways of life of the colonized. However, it should not be forgotten that the colonization practice has always been a contested matter, as the colonized groups have negotiated a space within this process. 

The history of food and eating habits in different contexts can help us to realize that methods of eating are inherently complex [8]. Since the time of liberation from the colonial powers, the culture of South Asia has coincided with the incorporation of a significant amount of cultural values transferred to them mainly from the British and the Europeans. After independence, the Sri Lankan government preferred to continue with the colonial economic structure. For example, urbanization has shown to have a positive effect on wheat consumption and a negative impact on rice consumption in Sri Lanka. Along with the growth of supermarkets, Sri Lanka has, over the last decade, observed a rapid increase in the number of Western fast food chains serving the big cities, which are increasingly spreading to smaller towns [7,9,10]. Even today, the influence of Europeans, though older than 500 years, is an integral part of the local culture of Sri Lanka.

Thus, after the Europeans left the South Asian region in the formal political sense, they supported their influence through neo-colonization followed by neoliberalism [11,12,13]. Some critics have argued that post-colonization is the continuation of colonization, in the sense the colonies get freedom only from the political rule. It is worth noting that an unhealthy diet is a trend dominating the health profile of an increasingly large number of people in developing, postcolonial countries [14]. In developing countries, poor people spend 80% of their income on food [15], yet they are often malnourished due to inadequate access to food [14,15]. Not only malnutrition, but also NCDs, such as cancer, occur as the Western diet displaces the traditional diet, due to the surplus of some harmful nutrients and the lack of some essential nutrients in Western foods. The world has reached a historic milestone. The “traditional” diet is used to mean food that is mainly plant-based, rich in grains, legumes, vegetables (plus their oils), and fruit, with little or no animal products. There is evidence that the traditional foods of Sri Lanka, including a broad range of indigenous cereals, roots, tubers, green leaves, fruits and vegetables, spices, animal fats, and fish, are linked with various health benefits, including protection from non-communicable diseases. The ‘Western’ diet is an evolving concept [15]. Today’s Western diet has adverse effects on health, equity, and the environment in Sri Lanka as can be seen in Sri Lanka’s food production and the population who consumes an increasingly Western diet [15].

The paper structure is as follows: Section 2 deals with the theoretical framework of the colonial and postcolonial dynamic and cultural change with respect to the nutrition transition in Sri Lanka. The following sections discuss the Sri Lankan traditional food system and how it changed in the colonial and postcolonial periods, including the main changes and their effects on current micronutrient deficiencies and non-communicable diseases. The paper also explains the factors that underpinned Sri Lanka’s changing diet, from early colonization to the post-colonialization period. 

## 2. Theoretical Framework

**Figure 1:**
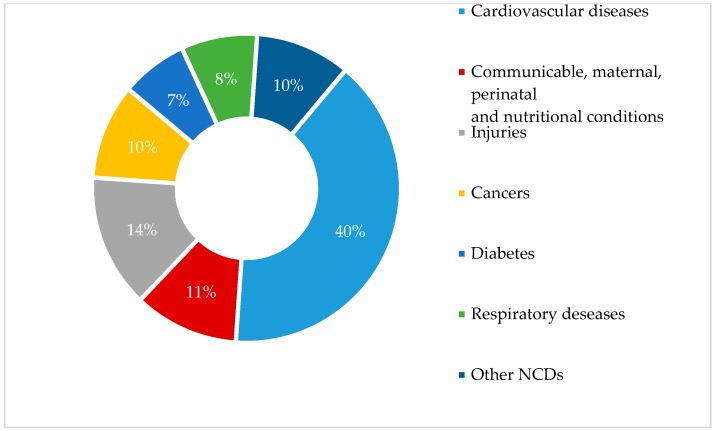
Percentage mortality by cause in Sri Lanka (2014). Source: World Health Organization [17]. NCDs: non-communicable diseases.

In this section, the conceptual framework is presented with ‘the colonial and postcolonial dynamics on food culture’ as the central approach. Based on the literature, the main themes of this discourse focus on food and the nutrition transition and their impact on human health. Human history is characterized by a series of changes in food and nutritional status. Throughout human existence, diet and nutritional status have undergone a series of major shifts among the broad patterns of food use, as reflected in changes in stature, body composition, and patterns of disease. Today, a marked worldwide shift towards a diet that is high in fat and processed foods and low in fiber, with corresponding increases in degenerative diseases, is evident [16]. NCDs are currently responsible for almost 70% of global deaths, the majority occurring in developing countries [17]. In indigenous communities, the nutritional change, characterized by the rapid Westernization of diet and lifestyle, is associated with an increasing prevalence of chronic diseases [18,19]. In Sri Lanka, approximately 75% of deaths in the country are caused by cardiovascular diseases, such as cancer, chronic respiratory diseases, diabetes, and other NCDs [17]. Figure 1 shows a downfall profile of deaths caused by diseases in Sri Lanka. It can be observed that 40% of deaths can be attributed to cardiovascular disease, followed by cancer, other NCDs, respiratory diseases, and diabetes.

Furthermore, contemporary food ways and identities are, in large measure, a product of a long history of colonial encounters [15]. Along with the loss of regional food itself, even more serious is the loss of traditional knowledge of production, harvesting, processing, preparation, and food uses the experience that sustained groups of people in their home region for thousands of years [18,19,20,21]. Colonialism has had a long-lasting impact on the lives of people [22]. The intimate links between food practices and the embodiment of identity between commensality and politics have made food a central arena for the development of colonial battles of several types. The fact is that colonialization had a direct impact on the cultural food habits in Sri Lanka. Therefore, a focus on food holds great analytical promise for archaeologists trying to understand ancient colonial situations and their transformative effects on identity [22,23]. Colonialism is the acquisition, establishment, maintenance, and expansion of authority over one area of people (the colony) by another people from a different area. The colonizers use the resources of the colonies and impose their culture on these colonies [24,25]. 

Colonialism has had a long-lasting impact on people’s lives [22]. Countries under colonial domination experience a distinctly separate way of developing and maturing, and ultimately, the inheritance of subsistence will continue to affect their survival and performance [21,25]. The situation becomes more complicated when the retreat of colonial rule does not lead to independence, but rather is replaced by another dominant power [26,27]. Even today, the impact of more than 500 years of influence by Europeans is an integral part of the local culture of Southeast Asia. Indeed, as noted above, after the Europeans left the South Asian region in the formal political sense, they supported their influence through neo-colonialism, followed by neoliberalism [28,29]. 

**Figure 2:**
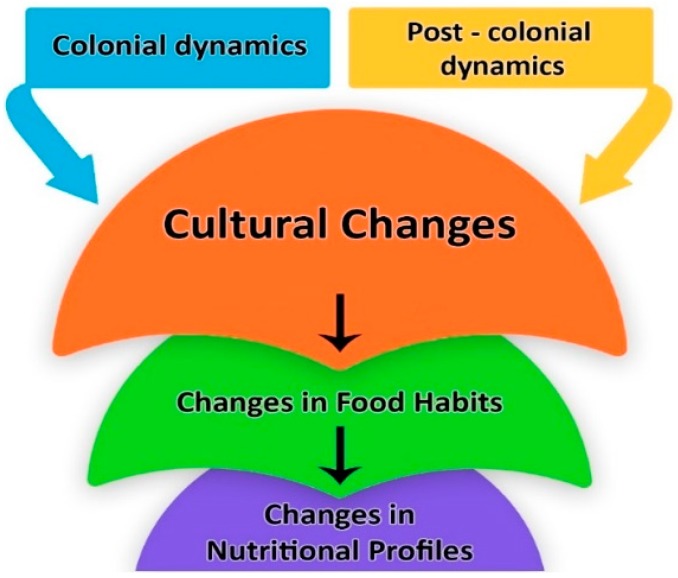
The conceptual framework for the colonial and postcolonial dynamics of food culture (authors’ illustration).

Over the centuries, the methods implemented to excise this indigenous knowledge have generally shifted from the use of overt force (e.g., slavery, religious conversion, seizure of arable land, and food supply) [30] to the implementation of a neo-colonial, political, and economic structure inherently designed to oppress through the creation of economic dependence [31]. However, there is limited scientific data about this factor. Still, it is essential to discuss traditional food culture and the changes in food culture after the colonization period. According to historical sources, modern research studies, and field interviews, it could be possible to identify two main groups that impact food habits and nutritional changes in this research study: colonial and postcolonial dynamics (Figure 2).

Colonialism should be understood as a dynamic intercommunicate in the context in which the colonial empires and individual colonies massively influenced the historical development of their European mother countries and vice-versa. Therefore, colonial and postcolonial dynamics directly impact cultural changes in colonies.

Based on the literature, different characteristics of cultural changes reflect colonial and postcolonial dynamics (Table 1). These characteristics either directly or indirectly impact cultural and food habit changes to the Western diet. During the colonial period, a colonial power was defined by the building and maintaining of a number of colonies in a territory by people from another region [32]. On the one hand the colonizers rejected cultural compromises with the colonized population [33,34]. On the other hand, in the post-colonization period, the former colonial powers and the colonized people had to learn to deal with a previous colonial society while still moving forward, which impacted cultural and food habit changes [34].

## 3. Materials and Methods

This research was conducted in two different marginal areas in Sri Lanka, a remote rural area in Kabithigollawa and an urban slum area in Colombo. Kabithigollewa is a village located in the north-central province whose residents engage largely in agriculture as their main source of income. The people of Kabithigollawa, along with the consequences of 30 years of war, suffer from poverty and malnutrition. This is also true, in particular, for the low-income residents living in the urban slum area of Colombo. These residents not only suffer from malnutrition but also other social and health-related problems. Historically, these issues can be seen as a result of urbanization alongside colonization. 

The research followed the integrated concept of ethnological and sociological study approaches. The study addressed a pragmatic and complex problem in which social factors played a role. The integrated design comprised of qualitative data as the primary source, while quantitative content analysis was used as the baseline data. Information and data were collected through field interviews, historical references, and modern research studies. Because oral history is a form of human communication, this research study conducted interviews and group discussions as the main qualitative data collection method for the primary data [42,43]. Oral history is a collection of stories and reminiscences of a person or persons who have first-hand knowledge of any number of experiences [44,45]. Therefore, the interviewers carried out discussions with older people over the ages of 80–110 years (*N* = 50) who had experience with the postcolonial period. The expert interviews were independent of each other in terms of the questions and area of the subject field. The experts were selected based on the nature of the research questions. Furthermore, there were open interviews and questions with local farmers. These were mainly incorporated to capture orally any historical production-related changes in the farming systems and dietary patterns in the dry zone and the urban area. The interview questions were slightly altered based on factors related to location but were always fixed according to the main thematic areas of the research. The gathered data from the field interviews were analyzed using descriptive statistics [46].

## 4. Results 

Based on the data, the results can be divided into two sections: ancient food culture and food transition influenced by colonization.

### 4.1. Ancient and Traditional Food Habits and Food Culture in Sri Lanka

When the agriculture is the cornerstone of Sri Lanka’s economy, with more than 70% of the population living in rural areas [47], the ancient concept of agriculture, especially rice farming, was based on “the tank, field, temple, and village” [48,49]. These four components of agrarian culture have been woven together to lead to prosperity. Food production was based not only on culture and religious rituals [49] but also on astrology and biotic and abiotic components in the environment [49]. In ancient Sri Lanka (before colonialization), there were no farmers as defined today, because people never owned farms or farming based on money. Agriculture was not a revenue-generating process, and at the same time, it was not considered to be a business or an industry. Agriculture was essentially everybody’s service, and it was the public’s responsibility to use and maintain the land for the sake of the nation [16]. That being said, Sri Lanka is an island with high biodiversity, and access to food was never a problem [47,48]. This is well documented in the old chronicles by Robert Knox (1681) and Emerson Tennent (1860). Most of the foods that the Sri Lankan elders enjoyed were not grown by them [27,50,51,52]. They were found everywhere, growing naturally. According to several interviews, during the post-colonization period, there were not only edible plants but also useful medicinal plants. Until the last few centuries, food was not produced for sale, but rather consumed and shared. In the Kebithigollewa area, most people had public lands called ‘Chena’, which were devoted to agricultural purposes [51,52]. There is evidence that the livelihoods of the old people came from the Chena cultivation, which was mainly done in the ‘*Maha* season’. The cultivation of Chena was based on the relocation of cultivated lands from one place to another. The primary farming methods were those that did not use irrigation methods or chemical fertilizers. The ‘Chena’ cultivation periodically reduced the number of trees of a small jungle land and set the woody growths on fire to create suitable land for cultivation. This virgin land was best suited for cultivation because of its rich soil. The farmers cultivated various crops in the Chena for the food needs of their families [53], such as ‘*tala*’(sesame), ‘*kurakkan*’ (finger millet), ‘*meneri*’ (millet), ‘*badairingu*’ (corn), ‘*mung*’ (green gram), ‘*bajiri*’ (*Echinichloa glabrescens*), and various varieties of vegetables. The most commonly cultivated nine varieties of foods were called ‘*nawadali*’ [54,55].

Chena farmers did not change their dwellings when they selected new lands, because they became sedentary as far as their homes were concerned. They built a hut in the ‘*chena*’ to protect the crops from wild animals, such as wild elephants, that roamed in search of food [55]. ‘Chena’ cultivators did not have the concept of private land ownership. Very often, these jungle lands belonged to the state, and the farmers who cleared the land, planted the crops, and got the produce for their labor did not seek to claim these lands for themselves. As was a common practice among Chena farmers, nobody had the right to plant a plot of land (Chena) that had been cleared by someone else [55,56]. 

According to the Department of Ayurveda’s data various research and oral interviews as shown in Table 2 and Table 3 some of the traditional Sri Lankan food had many nutritional benefits, and the Sri Lankan diet consisted of green leaves, which they used in many ways. In particular, green leaves were the main source of vitamins and other therapeutic values. Protein was mainly derived from vegetable sources, such as wing beans, velvet beans, drumsticks, kidney beans, and leafy vegetables with high protein content. Many vegetables were used for nutritional benefits and therapeutic values. Most leafy vegetables and legumes were collected from the home garden, rice fields, and nearby forests. There were many types of delicious mushrooms collected from the forest.

Individually, most of the food was obtained from natural sources and the majority of proteins from plant sources. The types of traditional foods, preparations, and consumption habits were more diverse than today. Traditional people consumed food not only for “nutrition” but also for “therapeutic purposes” (Table 2 and Table 3) [8,57].

They cultivated everything in their home gardens, including vegetables, fruits, spices, condiments, and even basic medicinal herbs for home remedies. The cooking oil was extracted from coconut (*Cocos nucifera*), *Mee (Madhuca longifolia*), *Sesame* (*Sesamum indicum*) [8,57,59]. They maintained a small backyard system that provided high-quality animal protein sources, such as eggs and milk. Meat was rarely eaten, and the animals that they raised were rarely slaughtered. Instead, Sri Lankan people ate “game meat” (wild meat), such as porcupine, jungle fowl, hare, wild boar, and so on. There was no shortage of any sources of “game meat” in the past, and the slaughtering of wild animals for meat (game) was allowed with restrictions. Game meat was appreciated less frequently. Some of the people reared goats, and goat milk was in high demand for domestic consumption. Goat’s milk was considered as a therapeutic food for allergies and asthma. As a result, goat’s milk is still in demand today. All these animal products were produced in the backyard without external input or expense. Important sources of animal protein were freshwater fish and milk, which were freely available. Consumption of fresh milk was not common among adults [8]. They understood that the nutrients derived from milk (protein and calcium) could also be obtained from vegetable sources. Therefore, they allowed the calf to drink its milk amply.

Different foods had distinctive and unique preparation systems. All the ingredients were natural, though some types of food were prepared only for a few occasions or purposes. Some types of food were specially prepared for special people. The food that was prepared and brought to the rice field to serve the people was called “*Ambula”* consisting of local vegetables and rice. The curries of *ambula* were made sour by using tamarind (*Siaymbala*) or garcinia (*Goraka*), in order to make it enriched with vitamin “C”. Another special preparation was sour fish curry (*Malu Ambul thiyal*), a unique spicy fish preparation with thick gamboge “*Goraka*” paste [8]. This shows, with diverse foodstuffs, how varieties of delicious dishes were prepared [8] Robert Knox recorded in his book, *An Historical Relation of the Island Ceylon in the East-Indies*, that this was relished in daily diets only by the noble people of Sri Lanka. The ordinary and the poor used them occasionally during special meals. The excess milk was always processed and conserved [8]. The most important processed and preserved dairy products were milk, cottage cheese, cream, whey, and ghee. These were popularly known as the five essential milk products. These five milk-based foods were considered to be noble foods.

Many traditional Sri Lankans have always been concerned about the type of food that they choose, including the quantity and the quality of their food. Food security and food availability in traditional Sri Lanka was so rich that it was consumed according to the type of person (child, adult, elder), physiology (sick, pregnant, nursing), degree of activity (less active, energetic), and the type of meal (breakfast, lunch, dinner). Rice was considered the staple food, as it was eaten three times per day. Various rice varieties were served to pregnant and lactating women, as well as to sick people and the monks [8,47]. For example, pregnant mothers were fed with ‘*maa wee*’ varieties, and small children and senior people who couldn’t easily digest other varieties were fed with “*heenaty*”. There are records that in ancient Sri Lanka, there were more than 2442 different varieties of rice [8,58]. 

They had detected compatible foods. Incompatible foods have always been avoided. If the food had any harmful effects, it was always omitted in an ordinary meal. For example, they did not drink milk but rather ate milk in the fermented form as curd. Today, scientific evidence has proven curd contains many beneficial bacteria. Cultivated and wild vegetables, especially wild green leaves and other wild plant food types were important ingredients for the sauces that accompanied the carbohydrate staples. The seeds were naturally hybridized and fertilized. The availability of food was plentiful. The choice of food was dependent on the need. For children to overcome the burden of intestinal worms, a ‘*mellun*’ prepared from ‘*Eth thora*’ *(Cassia alata*) or ‘*Erabadu*’ (*Erythrina indica*) was used; as for diabetic patients, a curry of bitter gourd (*Mormodia aurandica*) was given, Similarly, there were many other dietary recommendations that could be used for therapeutic and treatment purposes [8,57,61].

The data were collected by interviewing the senior members of the society in the research areas. According to these individual interviews, 96.8% of respondents mentioned that the vegetables they cooked were obtained from natural sources. The average age of the sample was 90 years old. This age group experienced the aforementioned social transitions and traditional cultural values during their lifespan. The villagers defined “naturally grown vegetables” as edible plants grown without special care without the use of pesticides, herbicides, and fertilizers and that are not grown using commercial seeds or for commercial purposes [8]. The vegetables were picked daily around the houses, preferably before cooking. They further mentioned that freshly picked vegetables made their food tastier. The villagers would share these food items, such as wild plants, mushrooms, and venison, in their hamlets when large amounts were collected.

### 4.2. Nutrition Transition after Colonization

Many developing countries are experiencing a rapid dietary change characterized by the double burden of diseases, in which chronic diseases are more common, while infectious diseases remain undefeated. Food habits in Asia began to shift dramatically at the onset of European colonial occupation. According to ancient literature, Sri Lanka had experienced the rule of three colonial nations: first, the Portuguese; then, the Dutch; and finally, the British. They introduced new aspects to and changed the food habits of the Sri Lankans.

During the Portuguese period, not only the above food types were introduced (Table 4), but also new food tastes, such as red chilies, were added in food preparations, which were not used very often in ancient Sri Lanka [64]. Sri Lankans had their supply of local varieties, which were in abundance and different. In the dry zone, people did not use peppers; rather, they used their own varieties of local and wild chilies. Then, the Dutch influenced the Sri Lankan culinary pattern. Lamprais is an enhanced version of traditional rice and curry [64]. The rice is boiled in stock and accompanied by *sambols* (hot chilies ‘Capsicum annum’ and coconut). Up to now *sambols* were more popular in every part of Sri Lanka. The product is moistened with coconut milk, wrapped in a banana leaf to enhance the flavor, and baked to produce a meal for special occasions [64]. Also, they introduced bread, variety of foods (Table 5) different types of cake with plums and sultanas, which were traditionally used at Christmas. *Kokis* (from the Dutch koekje,) is a crispy textured sweet made from rice flour and coconut milk, deep-fried in a wheel or flower shaped ‘‘mold’’, and until now, was eaten to celebrate Sinhala and the Tamil New Year. The simple stew also seems to have been introduced by the Dutch [64].

Sri Lanka’s traditional agricultural farming, food systems, and food culture were challenged for the first time during the British colonial period (19th century). The emphasis then was on plantation agriculture, which included the cultivation of tea, rubber, and coconut on a massive scale. The British brought South Indian Tamils to Sri Lanka to work as estate laborers. Thereafter, rice was also imported to fulfill the dietary requirements of their workers. As a result of the promotion of plantation agriculture, traditional agriculture was changed [65].

The unsuccessful *Uva Wellasa* uprising in 1918, just three years after the British captured power in Sri Lanka, also influenced the traditional agriculture adversely. The British rulers who managed to suppress the uprising nevertheless realized that the strength of the “rebels” lay in the prosperity of *Uva Wellassa* (“Wellassa” means one lakh of paddy fields) [66,67,68]. In order to prevent a further uprising, the present families were annihilated, the paddy fields and grain silos were burnt, and the tanks and other irrigation systems destroyed. In a short time, the whole of the *Magama* and *Uva Wellassa* regions were ruined [59]. The British did not encourage traditional farming for a considerable length of time, hoping to break the backbone of the traditional farmers. This seriously affected traditional food varieties and farming systems. Meanwhile, British colonialism destroyed the country’s biodiversity. Large forest areas were opened for monoculture and many new plant species were introduced into the country as crops [65]. The result of these practices led to gradual erosion of the island’s biodiversity. However, this condition did not affect the dry zone as they expected, and therefore, the biodiversity of the dry zone still remains rich. Today, because of “modernization”, most of the traditional food varieties have been lost, and many types of local fruits have been dried out [65].

European food habits (the Western diet) also had serious implications for Sri Lankan traditional food and farming systems. The Europeans introduced high intake of fats, salt, sugars, and processed foods, as well as food plants familiar to them in Europe, to satisfy their food requirements. Consequently, vegetables, such as cabbage, potatoes, carrots, beans, beetroot, and leeks, were grown in the hill country areas where the agro-ecological conditions are favorable for such crops [8]. The Sri Lankan elite imitated the British, and with time, these varieties of vegetables became popular among the common people as well. Even today, these vegetables are popularly known as “upcountry vegetables”, as they are cultivated in the highlands of Sri Lanka. For example, according to the experience of the elderly people in present Sri Lanka, some areas in Sri Lanka were promoted for some foods, such as wheat flour, tea, and alcohol by British people. In the past, people did not drink alcohol, and they did not have experience with alcohol other than ‘*Raa*’ (toddy made by coconut, palm/Palmyra). After the British had been expelled from the Sri Lankan subcontinent and also during their reign, the British not only influenced Sri Lanka politically, economically, and socially, but also influenced their spirit and culture to deep degrees. Besides, Western culture and its symbols are valued over native customs, leading to a kind of colonization in the mind. Still today, this colonization of the mind is one of the main causes of the identity crisis in Sri Lanka. It is accompanied by other factors, such as globalization, technological progress, and the disillusionment of youth with the indigenous powers and the increasing influences of Western culture. 

Globalization has been strongly associated with a significant increase in the concentration of corporate ownership across the entire food chain from production to processing, supply, and retail [7]. There is growing evidence that globalization and trade liberalization have played a key role in the dietary and nutritional transition in Sri Lanka. This includes food retail sectors and rapidly expanding supermarkets in Sri Lanka since the post-colonization period. Additionally, globalized food chains have the largest comparative advantage in supplying processed foods high in sugar, salt, and oil that are cheaper to produce and transport and have longer shelf lives than raw, unprocessed foods [7,9]. As a result, Sri Lanka has enjoyed a huge influx of processed food products, which have remarkably transformed the scenario of the food market and people’s food choices. The situation has changed. Supermarkets and groceries are taking the lead, selling cheap junk foods, such as cookies, chips and soft drinks.

## 5. Discussion

The impact of colonial dynamic and postcolonial dynamic on Sri Lankan culture, as well as cultural changes and their impact on food and nutritional changes, have been discussed. In ancient times, health conditions in the villages remained good due to the consumption of indigenous food and the use of indigenous medical methods [54]. The increase in food consumption has been accompanied by a change in dietary patterns. However, after the late 1970s, during the postcolonial period, this was used by literary critics to discuss the various cultural implications of colonization [21]. The consumption of indigenous traditional foods has also been used to prevent and cure many diseases. But, globalized food chains have the largest comparative advantage in supplying processed foods high in sugar, salt, and oil that are cheaper to produce and transport and have a longer shelf life than raw unprocessed foods [7]. As a result, Sri Lanka has enjoyed a huge influx of processed food products, which has remarkably transformed the scenario of the food market and people’s food choices. 

**Figure 3:**
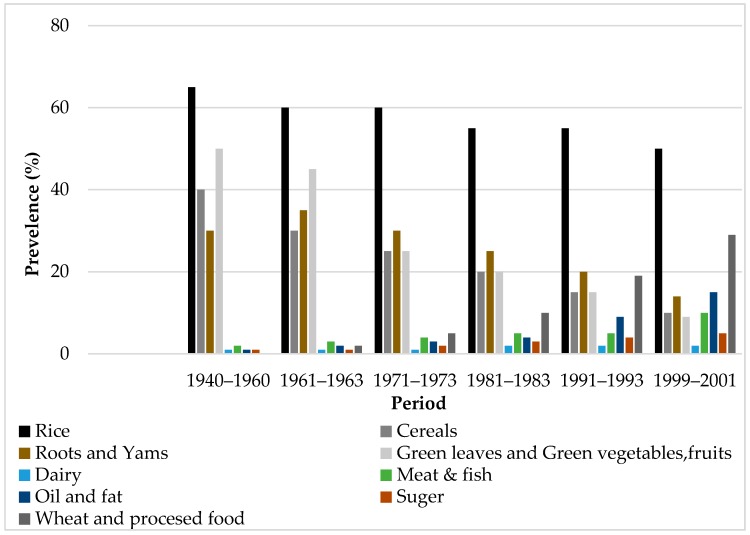
Growth in total food consumption in Sri Lanka. Source: Central Bank Reports from 1950 to 2001 [69].

According to Figure 3, Sri Lankans have recently spent more money on condiments and other food items, namely sauces, tinned or package stuff, soup cubes, chutneys, and prepared food bought from outside, than ever before. Over the period between 1940 and 2001, the consumption of traditional food varieties, such as root and yams, green leaves, and green vegetables, has decreased. It is believed that Sri Lanka was influenced mainly by fast food during the colonial era.

**Figure 4:**
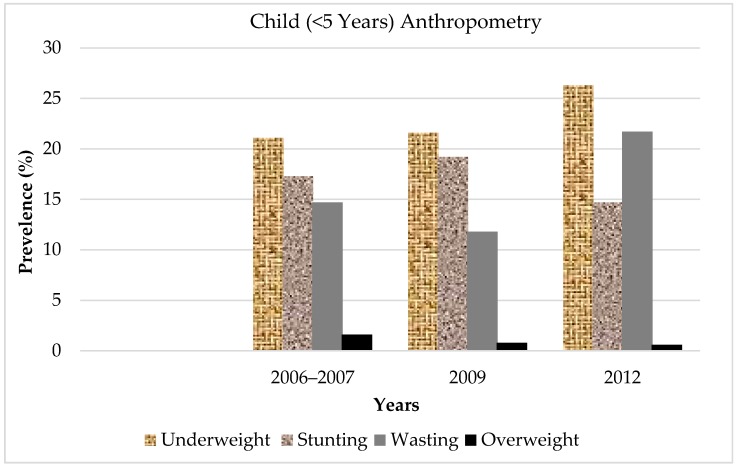
Child micronutrient deficiency in Sri Lanka. Sources: World Health Organization [71,72].

**Figure 5:**
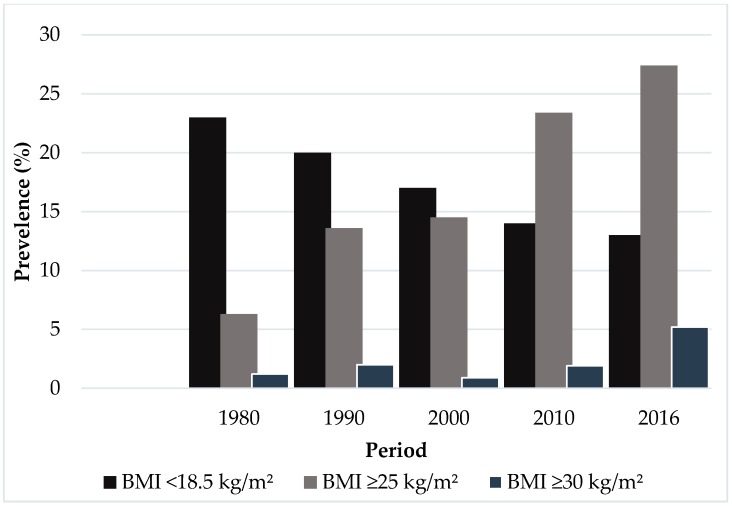
Female malnutrition based on body mass index (BMI) Sources: World Health Organization [71].

In 1902, Perera & Sons Bakers started its journey as a local fast food service provider in Sri Lanka [70]. After that, Royal Bakers and many others came to the area. Apart from local fast food providers, multinational fast food service providers started catering to Sri Lankan customers, such as McDonald’s (1998), KFC (1995), and Pizza Hut (1993) [70] with the advancement of globalization, facing what has become one of the biggest issues today relates to the link between child malnutrition and fast food. The reason for this is because postcolonialism represents and feeds an ideological response to the colonizer’s thoughts [34,36] regarding Sri Lankan culture. In addition, below, Figure 4 and Figure 5 help to clarify the child micronutrient deficiency and female malnutrition rates after the post-colonialization period. According to Figure 4, the number of children who are underweight and wasting (when part of the body becomes progressively weaker) increases steadily each year. Stunted growth and female malnutrition remain a big problem in Sri Lanka. Plant-based diets generally contain multivitamins, such as iron calcium, zinc, and vitamin A. Zinc deficiency contributes to stunted growth. According to a World Health Organization report, 4 mg of zinc can be met by traditional diets mixing whole grains, legumes, soy, and vegetables [15,71]. Therefore, it can be an effect of the traditional food diet change in Sri Lanka. The process of these nutrition changes and related health impacts have accumulated over a substantial period of time. Therefore, further research is required to deeply examine local communities to better understand these nutritional problems. The development and policing of the diet guidelines for traditional food in Sri Lanka is important for promoting healthy diets among the population. To be successful, the guidelines need to be understood and adopted by the majority of people in the country.

### 5.1. Destruction of the Traditional Farming Systems

In the past, Chena farmers cultivated a variety of crops to fulfill the food requirements of their families. After that, Chena farmers grew several crops specifically for the market in place of meeting the needs of their families [55]. Over the past three decades, Chena farmers have been trying to modify the Chena culture to make a profitable business in the market-driven economy. As a result, today’s Chena culture has deviated hugely from the traditional Chena culture. Thus, it clearly coincides with postcolonial dynamics that farmers now use machines to cut down the forests [55].

Even during postcolonial times, some state agencies used the land and directly influenced traditional farmers and the traditional varieties of food and seeds, which they used. As a government policy, a decision was made to allow the subsidization of fertilizers for the cultivation and purchase of Chena products at a high price on the open market, which reduced the organic fertility by more than 95% [73]. On the other hand, some farmers lost their cultivated areas due to the restriction of lands by the government to be used for development programs.

### 5.2. Disparities of Socioeconomic Status and Development of the Government Policy

Since independence, the manufacturing and service sectors have also developed with the food and agriculture sectors. However, the relative performance of each sector has been mixed; the manufacturing and service sectors have posted robust growth relative to the food and agriculture sectors [48]. There have been many significant changes in the structure of the Sri Lankan economy since the Western colonial powers ruled the country [48]. According to Munasinghe (2015) [74], this industrial booming in the colonial period is mainly due to the British change in social structure [74]. People from villages who were farmers have migrated to the Colombo to work in the harbor, railway, and other factories. This shift had a direct impact on the local social setting and the food system. Since the independence from British rule in 1948, several governments have taken many steps to promote activities in these two sub-sections (rice/other crops and plantations) in the areas of production, processing, and marketing. 

### 5.3. Creation of Cash-Crop Economies

The British exploitation of the 1950s led to the creation of “cash crop economies” in Sri Lanka. From the beginning, rural communities were encouraged by the British to grow food crops for export in order to earn enough money to improve their standard of living under a new economic system [75]. Nutritional habits were also radically altered by the introduction of new farming techniques that were assimilated into cash crop production. These techniques have favored the introduction of monocultures with higher yields of maize, rice, and other varieties. Monocultures have displaced traditional Sri Lankan food crops grown using traditional farming techniques, including “shifting cultivation” and “intermediate farming”, which have been historically adapted to local agricultural conditions. Traditional farming methods protected the soil, reduced weeds, provided communities with a variety of foods, and reduced the risk of crop failures, pests, and plant diseases. However, monocultures did not provide any of these benefits. The shift to monocultures and the reduction of dietary diversity have also resulted in a loss of knowledge about the old agricultural practices and traditional food varieties. 

### 5.4. Ecological Destruction

Various forest ecosystems were quickly cleared for growing crops. This ecological elimination has destroyed many native plant varieties and food varieties. Some native crops and wild food plants have been cut from traditional diets, which have affected the taste and nutritional value of ordinary dishes [75,76]. Monocultures have displaced the traditional crop cultivation methods, including shifting cultivation and intermediate crop production, to a commercialized system [7,76,77]. Overall, the introduction of new farming methods has brought economic benefits to the Western powers and caused incredible ecological destruction and human suffering in Sri Lanka. 

### 5.5. Nutrition-Related Propaganda (i.e., Advertising)

Marketing strategies are often deliberately tailored to existing cultural aspects regarding nutrition-related propaganda [78]. Clear contradictions and unusual connections are abundant in these advertising campaigns. For example, McDonald’s uses its resources and popularity to promote the United Nations Children’s Fund and its mission to eradicate child malnutrition [70]. This perhaps suggests that children should be “fed healthily” with McDonald’s food.

Advertising is now recognized as an important contributor to regime change and general acceptance of globalized food culture. Even today, mass marketing of packaged foods is ubiquitous, and the negative impact of these advertising campaigns is well documented. The best example of such propaganda was the mass marketing of and the sale of artificial milk powder. Many people in Sri Lanka sell their fresh milk and buy formula milk for infants, children, and the elderly. The use of milk formulas reduces the extent of breastfeeding and increases death rates caused by intestinal infections [1].

### 5.6. Disruption of the Family Unit

Economic reforms in Sri Lanka have disrupted the family unit by imposing higher pressure on women to work outside the house. Increasingly, women are forced to enter the urban labor market to improve the earning potential of the family [78,79]. This involves long hours of work to meet basic needs. The absence of women in family units has increasingly eliminated traditional foods that take a long time to prepare as compared with the preparation of imported cereals and high-calorie and nutrient-dense fast foods. With the entry of women into the economic employment sector, the breastfeeding of toddlers has declined. Reduced lactation periods are associated with poorer nutritional status and increased susceptibility to diseases, including diarrhea and measles in infants and children [72,73,74,75,76,77,78]. The consequences of the urbanization of Sri Lanka has led to a move away from high-fiber, home-cooked foods towards the consumption of a ready-made, prepared, packaged, and processed foods. The increased intake of *trans* fats, refined sugars, refined flours, and preservatives, and the low consumption of essential fibers and micronutrients have resulted in adverse effects on the health of the urban population. The extent to which global eating habits are being used in rural cities has not yet been studied effectively. However, recent evidence suggests that Western dietary habits are infiltrating rural Sri Lanka. Many urban residences are typically characterized by small living spaces, poorly equipped kitchens or outdoor cooktops, and limited access to natural energy sources and clean water, which disrupt traditional nutritional practices. Yet, there are still chances to develop and promote traditional dietary patterns for the Sri Lankan community [80].

## 6. Conclusions

Over the past 400 years, multiple regime changes have taken place in Sri Lanka, which have been driven by both open and covert methods of control from colonization to the postcolonial period. But during that colonization period, both indigenous knowledge and the environment were destroyed and suppressed until the country’s independence, presenting economic independence; yet, even after independence, neocolonialism caused further destruction of Sri Lanka’s traditional culture and landscape. Forced rapid urbanization contributed to the destruction of the family unit and the introduction of a more globalized food system. All in all, colonial and postcolonial dynamics had a significant impact on cultural changes. This has directly impacted Sri Lankan food culture and nutrition status and is related to current nutrition problems. However, the rapid transformation of diets and changes in food systems at all levels (production, processing, distribution, and retail) pose several important additional challenges to food security and food policy, smallholder welfare, and agricultural research and development priorities. Sri Lanka is observing a dramatic transformation in its food supply systems in response to rapid urbanization, dietary diversification, and liberalization of foreign direct investment in the food sector. Supermarkets tend to replace central food markets, neighborhood stores, and street food sellers in urban areas. The observed changes are in the retail sector, as well as in the production sector. Supermarkets and fast food chains arise from and reinforce the Westernization of demand that results from economic development and urbanization. Changes in traditional diets towards a more Western diet, promoting as higher fat and sugar content, are expected to result in higher incidence of dietary-related non-communicable diseases, as well as micronutrient deficiencies. Thus, the dissemination of traditional knowledge and popular campaigns must continue. Ultimately, the individual, the family, and the local community must come together to regain their birthrights, so that they can grow and consume their local foods according to their traditional practices. In this study, it was identified that the food transition and socioecological patterns are important factors in the politics of policy-making. Dietary changes associated with urbanization are related to the fact that rural dwellers tend to be more self-reliant in obtaining food and also tend to eat traditional diets that are high in grain and fruit and vegetables and low in fat. In summary, it must be said that more must be done to expose the colonial and postcolonial forces that have undermined food security in Sri Lanka.

## Figures and Tables

**Table 1:** Colonial and postcolonial dynamics.

Period	Dynamics
Colonial	Policy and practice of power/control over weaker peoples or areas [31]
	The system or policy of a nation seeking to extend or retain its authority over other people or territories [32]
	Contributions to colonial aspirations of power and control over the territory; the government response was to impose environmental and social control [33]
	Cultural superiority [34]
	Colonial powers into the culture [35]
	Control by one power over dependent areas or peoples [36,37]
	Invest their identity to the colony [38]
	The colonizers are convinced of their greatness and their ordained mandate to rule [39]
Postcolonial	Postcolonialism represents an ideological response to colonialist thought [40]
	Moving toward the development of a more cross-culturally oriented system [41]
	Dealing with previously colonized societies [41]

**Table 2:** Traditional wild fruit in Sri Lanka.

Vernacular Name	Botanical Name	Food Use	Nutritional and Therapeutic Value	Edible Parts
*Katu-attha/Weli-attha (Annona)*	*Annona muricate/Annona reticulata*	Ripened fruits was eaten fresh	Leaf infusion used as sudorific; antispasmodic; emetic flowers are antispasmodic. The ripened fruit is antiscorbutic; the unripe fruit was used for dysentery. Fresh leaves were used as topicals, applied to the stomach of children suffering from indigestion	Fruit
*Thal* Palmyra Palm	*Borassus flabellifer* L.	The inflorescence is tapped for toddy, vinegar, and jaggery. Young nut water (liquid endosperm) was drunk.	Ripened fruit is rich in vitamins A and C. The toddy is beneficial for inflammatory ailments and dropsy. It is a diuretic prescribed for chronic gonorrhoea and amoebiasis.	Fruit and germinating seed root
*Divul* (Wood Apple)	*Feronia limonia* L.	Mature and ripe fruit was eaten fresh and drunk	The pulp of the unripe fruit along with other ingredients were used for chronic diarrhea and dysentery. The ripe fruit was useful in hiccups and ailments of the gums and throat and was applied externally on bites of venomous insects.	Fruit
Bel fruit (Slime Apple)	*Aegle marmelos* (L.) *Corrêa*	Ripened fruits were eaten fresh. The shell and flowers drunk were as a beverage.	This fruit used for fever, hypochondria, melancholia, palpitation of the heart, diarrhea, and gastric troubles in children. The leaves were given for jaundice and anasarca.	Fruit and flower

Sources: [57,58,59,60,61,62,63].

**Table 3:** Traditional food items in ancient Sri Lanka.

Food Varieties	Vernacular Name	Botanical Name	Nutritional Value
*Yams*	*Raja ala* (Greater yam)	*Dioscorea alata*	Moisture 76 g, Energy 87 kcal, Protein 1.9 g, Fats 0.2 g, Carbohydrates 20 g, Calcium 38 mg, Iron 1.9 mg
*Green leaves*	*Heen-gotukola*	*Centella asiatica*	Moisture 84.5 g, Energy 37 kcal, Proteins 2.1 g, Fats 0.5g, Carbohydrates 6.0 g, Calcium 224 mg, Phosphorus 32 mg, Iron 68.8 mg
	*Pethi-thora* (Fetid cassia)	*Cassia tora*	Moisture 85.7 g, Energy 45.0 kcal, Protein 4.0, Fat 0.5 g, Carbohydrates 6.1 g, Calcium 397.0 mg, Iron 83.0 mg, Carotene 25.0 meg, Vitamin C 99.0 mg
	*Heen sarana* (Horse purslane)	*Trianthema portulacastrum*	Moisture 93.4 g, Energy 21.0 kcal, Protein 2.1 g, Fat 0.3 g, Carbohydrate 2.3 g calcium 50 mg, Phosphorus 28 mg, Iron 2.4 mg, Vitamin A 495 mg
	*Iramusu*	*Hemidesmus indicus*	Moisture 92.1 g, Energy 26 kcal, Proteins 2 g, Fats 0.7g, carbohydrates 2.9 g, Calcium 73 mg, Phosphorus 21 mg, Fe 10.9 mg, Carotene 5.586 mg
	*Kathuru-murunga*	*Sesbania grandiflora*	Moisture 73.1 g, Energy 93 kcal, Proteins 8.4 g, Fats 1.4 g, Carbohydrates 11.8 g, Calcium 1130 mg, Phosphrus 80 mg, Iron 3.9 mg, Carotene 5400 meg, Vitamin C 169 mg
*Cereal*	*Undu* (Black gram)	*Phaseolus mungo*	Moisture 10.9 g, Energy 347 kcal, Proteins 24 g, Fats 1.4 g, Carbohydrates 59.6 g, Calcium 154 mg, Phosphrus 385 mg, Iron 9.1 mg, Carotene 38 g
	*Kollu* (Horse gram)	*Dolichos biflorus*	Moisture 11.8 g, Energy 321 kcal, Proteins 22 g, Fats 0.5 g
	*Mun-eta* (Green gram)	*Phaseolus aureus*	Moisture 10.1 g, Energy 348 Kcal, Proteins 24.5 g, Fats 1.2 g, Carbohydrates 59.9 g, Calcium 75 mg, Phosphorus 405 mg, Iron 8.5 mg, Carotene 49 meg

Sources: [58,59,60,61,62,63,64].

**Table 4:** New variety of food introduced by Portuguese.

Vernacular Names	Botanical Name	Edible Parts	Distribution	Therapeutic Value
*Emberella.*	*Spondias pinnata*	Fruits	Native of Polynesia	The juice of the leave is used for earaches. The fruit is an antiscorbutic, and the acidic and astringent pulp is used for bilious dyspepsia.
*Katu-attha*, (Soursop)	*Annona muricata*	Fruits	Native of the west Indies	Leaf infusion is used as sudorific; antispasmodic; emetic flowers are antispasmodic. The ripe fruit is antiscorbutic; the unripe fruit is used for dysentery.
*Annasi*, (pineapple)	*Ananas comosus*	Fruits	Tropical America	Good source of vitamins A, B, and C and calcium and iron. The fruit juice of the leaves was a powerful anthelmintic and vermicide.
*Mangnokka, Maiyokka*, (Cassava, Manioc)	*Manihot esculentus*	Roots and young leaves	Mexico and parts of Guatemala, northeastern Brazil	Pounded leaves are applied as a compress to the head in fevers and headaches. A decoction of the bark of the trunk is considered antirheumatic. Bark decoction is anthelmintic.
*Miris, Malu miris*, (Chilly, Capsicum)	*Capsicum annum*	Fruits	Central and South America	The fruits are acrid, bitter, thermogenic, digesting carminative, laxative, expectorant, sialagogue, stimulant, and cardiotonic.

Sources: [57,58,59,60,61,62,63].

**Table 5:** A new variety of food introduced by the Dutch.

Vernacular Names	Botanical Name	Edible Parts	Distribution	Nutritional and Therapeutic Value
*Bathala*, (Sweet potato)	*Ipomoea batatas*	Roots and tender leaves	Endemic to Central America	Sweet potato tops, particularly the purplish ones, are used for diabetes. The leaves are applied for boils, carbuncles, and pimples. Boiled sweet potato is good for diarrhea.
*Gadu guda*,	*Baccaurea metleyana*	Fruits	Southeast Asia.	-
*Bada iringu*, (corn, maize)	*Zea mays*	Grains	South America	-

Sources: [57].
